# Phenotypic and molecular characterization of carbapenem resistance among clinical isolates of *Pseudomonas aeruginosa* and *Acinetobacter baumannii*

**DOI:** 10.1186/s12866-026-05377-x

**Published:** 2026-07-08

**Authors:** Gopiram Syangtan, Laxmi Kant Khanal, Shrijana Bista, Arun Bahadur Chand, Prabin Dawadi, Binod Rayamajhee, Reshma Tuladhar, Shiba Kumar Rai, Dev Raj Joshi

**Affiliations:** 1 Shi-Gan International College of Science and Technology (SICOST), Tribhuvan University, Kathmandu, Bagmati Nepal; 2 Central Department of Microbiology, Tribhuvan University, Kathmandu, Bagmati Nepal; 3 Department of Microbiology, Nepal Medical College and Teaching Hospital, Kathmandu University, Kathmandu, Bagmati Nepal; 4 Department of Microbiology, KIST Medical College and Teaching Hospital, Lalitpur, Bagmati Nepal; 5 Faculty of Medicine, Health and Human Sciences, Macquarie University, Macquarie, Australia; 6 School of Optometry and Vision Science, Faculty of Medicine, UNSW, Sydney, Australia; 7 Department of Infection and Immunology, Kathmandu Research Institute for Biological Sciences (KRIBS), Lalitpur, Bagmati Nepal

**Keywords:** Carbapenem resistance, ESBLs, MBL, *P. aeruginosa*, *A. baumannii*, Co-occurrence, Nepal

## Abstract

**Background:**

Carbapenem-resistant *Pseudomonas aeruginosa* (CRPA) and *Acinetobacter baumannii* (CRAB) were listed on the WHO bacterial priority pathogens list in 2017. These gram-negative bacteria often cause healthcare-associated infections (HAIs), which pose an increasing threat to public health. This study aimed to isolate and identify CRPAs and CRABs from a tertiary care hospital in central Nepal and characterize carbapenem resistance genes at the molecular level.

**Materials and methods:**

A total of 59 isolates, including 27 *Pseudomonas aeruginosa* and 32 *Acinetobacter baumannii*, were collected from different clinical samples from April 2021 to December 2022. Antibiotic susceptibility was determined via the Kirby disc diffusion method. The modified carbapenem inactivation method (mCIM) was used for phenotypic confirmation of carbapenem resistance. Conventional polymerase chain reaction (PCR) was used to detect carbapenem resistance genes.

**Results:**

Among the 59 isolates, 76.3% were multidrug resistant (MDR), 63.0% were extended-spectrum beta-lactamase (ESBL), and 62.7% were carbapenem resistant. Among the carbapenem-resistant isolates (*n* = 37), 75.7% of metallo-beta-lactamase (MBL) producers and 70.3% carried at least one carbapenem resistance gene. The *bla*_CTX−M_ gene was predominant among the ESBL-positive isolates, present in 58.8% of the *P. aeruginosa* isolates and 87.5% of the *A. baumannii* isolates, whereas 29% of both isolates harbored the *bla*_TEM_ gene. Among the CR-PA isolates, 58.3% carried *bla*_VIM−2_, 16.7% carried *bla*_NDM−1_, and 8.33% carried *bla*_OXA−23_. Among the CR-AB isolates, 56% harbored *bla*_OXA−23_, 32% carried *bla*_NDM−1_, 16% had both *bla*_NDM−1_ and *bla*_OXA−23_, and 8% contained both *bla*_OXA−58_ and *bla*_OXA−23_.

**Conclusion:**

This study highlights a significant burden of MDR and carbapenem-resistant *P. aeruginosa* and *A. baumannii*, with the co-occurrence of carbapenem resistance genes, in a clinical setting in Nepal. Our findings suggest that active surveillance of antibiotic resistance in pathogens could inform clinicians and healthcare providers about the treatment of infections.

**Supplementary Information:**

The online version contains supplementary material available at https://doi.org/10.1186/s12866-026-05377-x.

## Introduction

*Pseudomonas aeruginosa* and *Acinetobacter baumannii* are included in the ESKAPE (*Enterococcus faecium*, *Staphylococcus aureus*, *Klebsiella pneumoniae*, *A. baumannii*, *P. aeruginosa*, and *Enterobacter species*) group of pathogens identified by the World Health Organization (WHO) as critical-priority pathogens [1], indicating urgent interventions with novel antibiotics. These are non-lactose-fermenting gram-negative bacteria (NLF-GNB) and are epidemiologically significant as opportunistic pathogens [2], which often cause hospital-acquired infections (HAIs), such as catheter-associated urinary tract infections, surgical site infections, ventilator-associated pneumonia, central line-associated bacteremia, septicemia, meningitis, malignant external otitis, intra-abdominal infection, and wound infections [3]. The ability of *A. baumannii* and *P. aeruginosa* in a hospital environment enhances antimicrobial resistance, posing a notable mortality threat, especially in developing countries such as Nepal [4]. Infections caused by these bacteria pose a treatment challenge because of their emerging intrinsic and acquired resistance to different classes of antibiotics worldwide [5]. Carbapenem, a broad-spectrum beta-lactam antibiotic, is frequently used as a “last-line” drug of choice for the empirical treatment of critically ill patients infected with these bacteria [6].

Over the past two decades, the burden of antibiotic resistance related to multidrug-resistant (MDR) bacteremia has increased substantially in Nepal due to the widespread use of antibiotics [7, 8]. Previous reports have shown that last-resort antibiotics such as carbapenems and polymyxin are used excessively in low- to middle-income countries (LMICs) in healthcare settings [9]. Recent studies have shown high carbapenem resistance among *E. coli*, *K. pneumoniae*, *A. baumannii*, and *P. aeruginosa* in Southeast Asia, including Nepal [6, 10, 11].

A recent study in Nepal reported that more than 75% of *P. aeruginosa* and *A. baumannii* are phenotypically carbapenem-resistant [12, 13]. Additionally, these pathogens are highly resistant to multiple antibiotics due to intrinsic and acquired resistance mechanisms, including biofilm formation, enzymatic inactivation, porin loss, and efflux pump formation. Beta-lactam hydrolyzing enzymes such as Ambler class D (oxacillinase-23, 24, 58, and 143), Ambler class B (metallo-beta-lactamases (MBLs): IMP, VIM, NDM, SPM, GIM, and SIM), and Ambler class A (extended-spectrum beta-lactamases (ESBLs): TEM, SHV, and CTX) are responsible for antibiotic resistance in these bacterial isolates [2].

Coinfection by these pathogens leads to severe disease, leading to deadly outcomes [4]. Moreover, the prevalence of these two pathogens and their antibiotic resistance is greater in Nepal [12, 13]. This highlights the importance of focusing on *P. aeruginosa* and *A. baumannii* from the ESKAPE group because of their high resistance, nosocomial prevalence, and clinical severity. However, few studies have reported carbapenemase-associated genes in CR-PA and CR-AB isolates in this region [14, 15]. Extensive information on the distribution of carbapenemase enzymes and associated genes in CR-PA and CR-AB within clinical settings remains limited. In particular, the distribution of major resistance genes and their correlation with phenotypic resistance patterns remains insufficiently characterized. Our study aimed to identify phenotypes and genotypes to characterize carbapenemase-producing *A. baumannii* and *P. aeruginosa* from a tertiary care hospital in central Nepal. Genotypic testing is essential for accurate antibiotic susceptibility results, as it reveals the prevalence and types of carbapenemase-encoding genes carried by phenotypically antibiotic-resistant bacteria in the clinical setting [9]. The findings of this study might aid in understanding local antibiotic resistance surveillance and genetic epidemiological data for antibiotic stewardship programs and in developing a new strategic action plan for the treatment of MDR and carbapenem-resistant isolates for clinicians or healthcare providers in Nepal.

## Materials and methods

### Ethical considerations

Ethical approval (IRC no. 077/78/54) was obtained from the Institutional Ethical Review Committee of the KIST Medical College and Teaching Hospital, Tribhuvan University. Informed consent was obtained from each participant after clearly mentioning the benefits and possible outcomes of this study. After obtaining consent, demographic information was recorded. The personal details of the participants were kept confidential and archived.

### Study design, site, and period

A hospital-based cross-sectional study was conducted from April 2021 to December 2022 at the KIST Medical College and Teaching Hospital (KMCTH) located in Lalitpur and the Central Department of Microbiology, Tribhuvan University, Nepal. The clinical specimens were collected and processed at the microbiology and molecular laboratory, KMCTH, for bacterial isolation and identification. The isolates identified as *P. aeruginosa* and *A. baumannii* were further investigated for carbapenem resistance phenotypically and genotypically at the Central Department of Microbiology, Tribhuvan University.

### Study population and Sample collection

The study population was considered from inpatients and outpatients visiting KMCTH, including all age groups and both genders. A total of 1207 non-duplicated clinical samples, including wound swabs/pus, sputum, urine, blood, tracheal aspirates, and catheter tips, were randomly collected aseptically in clean, sterile, wide-mouth and leak-proof containers, appropriate for each specimen type, following standard microbiological guidelines. The properly labeled sample with no visible signs of contamination was accepted; otherwise, a repeat sample was requested. Patient samples with incomplete demographic information, unlabeled or duplicated samples, and contaminated samples were excluded from the study.

### Specimen culture for bacterial isolation and identification

All clinical specimens were inoculated on blood agar, MacConkey agar, and chocolate agar (Himedia Laboratories, India) for bacterial isolation, which was subsequently incubated for 24 h at 37 °C. The blood specimens were collected under aseptic conditions and diluted in BHI broth in a ratio of 1:10 and thoroughly mixed to ensure homogeneity. The BHI broth was incubated at 37 °C for 96 h, with subcultures transferred onto blood agar, chocolate agar, and MacConkey agar plates at 24-hour intervals. Blood agar and chocolate agar plates were incubated in a 5–10% CO₂ containing incubator for 24 h [16, 17]. After incubation, colony morphology characterization, Gram staining, and biochemical tests were performed to identify the isolates. Furthermore, *P. aeruginosa* and *A. baumannii* must be confirmed via special characteristic tests.

In brief, *P. aeruginosa* isolates were identified as non-lactose fermenting, grew aerobically on MacConkey agar, gram-negative, slender rods that exhibited motility, catalase, and oxidase, both positive, citrate-positive, alkaline/alkaline reaction without H₂S production on Triple Sugar Iron (TSI) agar, urease-negative, thrived at 42 °C, and were greenish pigmented on cetrimide agar (Himedia Laboratories, India) [18].

Similarly, *Acinetobacter* spp. were initially identified as non-lactose-fermenting, gram-negative cocobacilli, catalase-positive, oxidase-negative, non-motile, indole-negative, citrate-positive, urease-negative, alkaline/alkaline reaction without H₂S production on Triple Sugar Iron (TSI) agar, with aerobic growth on MacConkey agar at 44 °C [19]. Further, genotypic identification of *A. baumannii* was subsequently performed by detecting the *bla*_OXA−51_ gene, an intrinsic marker of *A. baumannii* [19].

Pure cultures of *P. aeruginosa* and *A. baumannii* isolates were preserved in tryptic soy broth (TSB) supplemented with 40% glycerol until further experiments and stored at -80 °C.

### Antimicrobial susceptibility testing

To test the antimicrobial susceptibility patterns of the isolates, the Kirby–Bauer disk diffusion method was performed on Mueller–Hinton agar (Hi-media Laboratories, India), and the results were interpreted according to CLSI (2020) [20]. The following antimicrobial discs were used for breakpoint determination: piperacillin (PI; 100 µg), piperacillin/tazobactam (PIT; 100/10 µg), ceftriaxone (CTR; 30 µg), ceftazidime (CAZ; 30 µg), cefotaxime (CTX; 30 µg), cefepime (CPM; 30 µg), aztreonam (AT; 30 µg), imipenem (IMP; 10 µg), meropenem (MRP; 10 µg), amikacin (AK; 30 µg), gentamicin (GEN; 10 µg), and ciprofloxacin (CIP; 5 µg). (Himedia Laboratories, India). We considered MDR isolates that were resistant to two or more antibiotics from different classes, such as beta-lactam, β-lactam combination, aminoglycoside, carbapenem, and fluoroquinolone antibiotic classes [14].

### Phenotypic detection of ESBLs

The isolates that were resistant to cefotaxime (30 µg) and/or ceftazidime (30 µg) were tested for ESBL production via combined disc methods involving the use of ceftazidime (30 µg) and ceftazidime/clavulanic acid (30 µg/10 µg) discs and cefotaxime (30 µg) and cefotaxime/clavulanic acid (30 µg/10 µg) discs. A disc was placed 20 mm apart, and a zone of ≥ 5 mm in the cefotaxime or ceftazidime with clavulanic acid disc compared with the cefotaxime or ceftazidime disc alone was considered positive for ESBL production after overnight culture on MHA plates [20].

### Phenotypic detection of carbapenem-resistant

Bacterial isolates that showed intermediate resistance or resistance to imipenem or meropenem were suspected to be carbapenemase producers [21]. The modified carbapenem inactivation method (mCIM) was employed to detect carbapenemase production in isolates. An ethylenediaminetetraacetic acid (EDTA) synergy test was used to confirm metallo-β-lactamase production.

Briefly, a 10-µL loopful of test organism was obtained from an overnight culture on a BA plate and mixed into 2 mL of tryptic soy broth (TSB). The mixture was vortexed for 10–15 s, and a 10-µg meropenem disk was added to each tube using sterile forceps, ensuring the entire disk was immersed in the suspension. Incubation was carried out at 37 °C for 4 h ± 15 min. Meanwhile, a 0.5 McFarland turbidity solution of *E. coli* ATCC 25,922 was prepared in NB. The MHA plate was then inoculated with *E. coli* ATCC 25,922 following standard procedures. The plates were allowed to dry for 5–10 min before the meropenem disks were added. The meropenem disk from each TSB meropenem disk suspension was slowly extracted. The flat side of the loop was pressed against the edge of the disk to leverage surface tension and extract the disk from the liquid. The loop was then gently dragged and compressed along the inside edge of the tube to clear any excess inoculated media from the disk before placing it on the MHA plate inoculated with the meropenem-susceptible *E. coli* ATCC 25,922 indicator strain. Up to four disks were placed on a 90 mm MHA plate. The MHA plates were incubated at 37 °C for 18–24 h, after which the inhibition zones were analyzed according to standard disk diffusion methods [22]. For screening of MBL, ethylenediaminetetraacetic acid (EDTA) was used for synergistic effects with imipenem discs [imipenem and imipenem + EDTA (10 µg/750 µg)], and the zone of inhibition was compared with that of imipenem alone for the screening of MBL producers [23].

### MIC test for carbapenem

The minimal inhibitory concentration (MIC) of a carbapenem (meropenem) for *P. aeruginosa* and *A. baumannii* was determined via the agar dilution method [20]. The test was performed independently in duplicate samples, and discrepant results were repeated. Briefly, a twofold dilution series of meropenem at different concentrations (0.5–1024 µg/mL) was incorporated into MHA plates. Bacterial suspensions were prepared from fresh overnight pure cultures and adjusted to a 0.5 McFarland standard. Then, bacterial suspension was diluted to a 1:10 ratio in NB and spotted 1 µL onto each of the meropenem-containing agar plates, and incubated aerobically at 37 °C for 24 h. The *E. coli* ATCC 25,922 was included in each testing batch as a quality control strain, whereas the negative control was considered the only culture medium used to ensure sterility. After 24 h of incubation at 37 °C, the MIC readings were recorded and interpreted according to the CLSI M100 [20]. The results were accepted only when MIC values fell within CLSI-recommended ranges for the quality control strain. Isolates with carbapenem MICs of ≥ 8 µg/mL were considered resistant, those with MICs of 4 µg/mL were considered intermediate, and those with MICs of ≤ 2 µg/mL were considered susceptible [20].

### Molecular detection of ESBL- and carbapenemase-encoding genes

The extraction of plasmid DNA from bacterial cultures in the logarithmic growth phase was conducted via the phenol: chloroform method [24]. The quality and purity of the extracted DNA template were assessed using NanoDrop measurements (NanoDrop, Thermo Scientific, USA).

The single-plex PCR assay was carried out via a thermal cycler (Bio-Rad, USA) with primer pairs (Macrogen, Korea) for *bla*_CTX−M_, *bla*_TEM_, *bla*_NDM−1_, *bla*_VIM−2_, *bla*_OXA−23_, *bla*_OXA−51_, and *bla*_OXA−58_ to detect beta-lactamase genes [] (Supplementary information, S1 Table). The reaction was carried out in a total volume of 25 µL (12.5 µL of 1X FIREpol Master Mix (Solis Biodyne, Estonia), 3 µL of template genomic DNA, 3.0 µL of 10 picomole primers (1.5 µL of forward primer and 1.5 µL of reverse primer), and 6.5 µLof nuclease-free water). The PCR products were visualized by agarose gel electrophoresis (1.5%) with DNA-stained safe dye (Sigma–Aldrich, Germany) at 70 V for 50 min. The amplified ESBL and carbapenemase genes were visualized in a gel documentation system (Azure Biosystems, USA), and band sizes were compared along with a 100 bp DNA ladder (Thermo Scientific, USA).

Previously confirmed carbapenemase- and ESBL-producing isolates were used as positive controls for PCR amplification, including *bla*_NDM−1_, *bla*_OXA−23_, *bla*_OXA−51_, and *bla*_OXA−58,_ producing *Acinetobacter baumannii* strains provided by Prof. Dr. Balaji Veeraraghavan, Christian Medical College, Vellore, India [25]; *bla*_VIM−2_, producing *Pseudomonas aeruginosa*, provided by Anil Shah from Annapurna Neurological Institute and Allied Sciences, Kathmandu, Nepal [15] and *bla*_TEM_ and *bla*_CTX−M_ genes producing clinical isolates of *E. coli*, maintained in our department [26] were used as positive controls, and nuclease-free water was used as a negative control in each PCR run.

### Quality control

The sterility of freshly prepared culture media was confirmed by incubating 5% of the plates at 37 °C for 24 h to check for potential contamination. All antibiotic discs and Mueller-Hinton agar (MHA) were inspected for manufacture, expiration date, and lot number, and stored under appropriate conditions. The quality of antibiotic disks was assessed with *E. coli* ATCC 25,922 by the AST method.

### Data analysis and interpretation

All the study data were entered into the worksheet of the Statistical Package for Social Science (SPSS) Software (Student version) and were used to analyze the data. The data and results were presented using percentages, rates, and inferential statistics. Comparative analyses were performed on bacterial species with specimen type, hospitalization status (inpatient/outpatient), age group, gender, and resistance phenotypes (ESBL, MBL, and carbapenem resistance) to identify factors potentially associated with antimicrobial resistance and to describe the rate of resistant isolates. Associations between categorical variables were assessed using Pearson’s Chi-square test or Fisher’s exact test with 95% confidence intervals (CIs). *p*-values *≤* 0.05 were considered statistically significant. The figure was created via GraphPad Prism software.

## Results

**Fig. 1:**
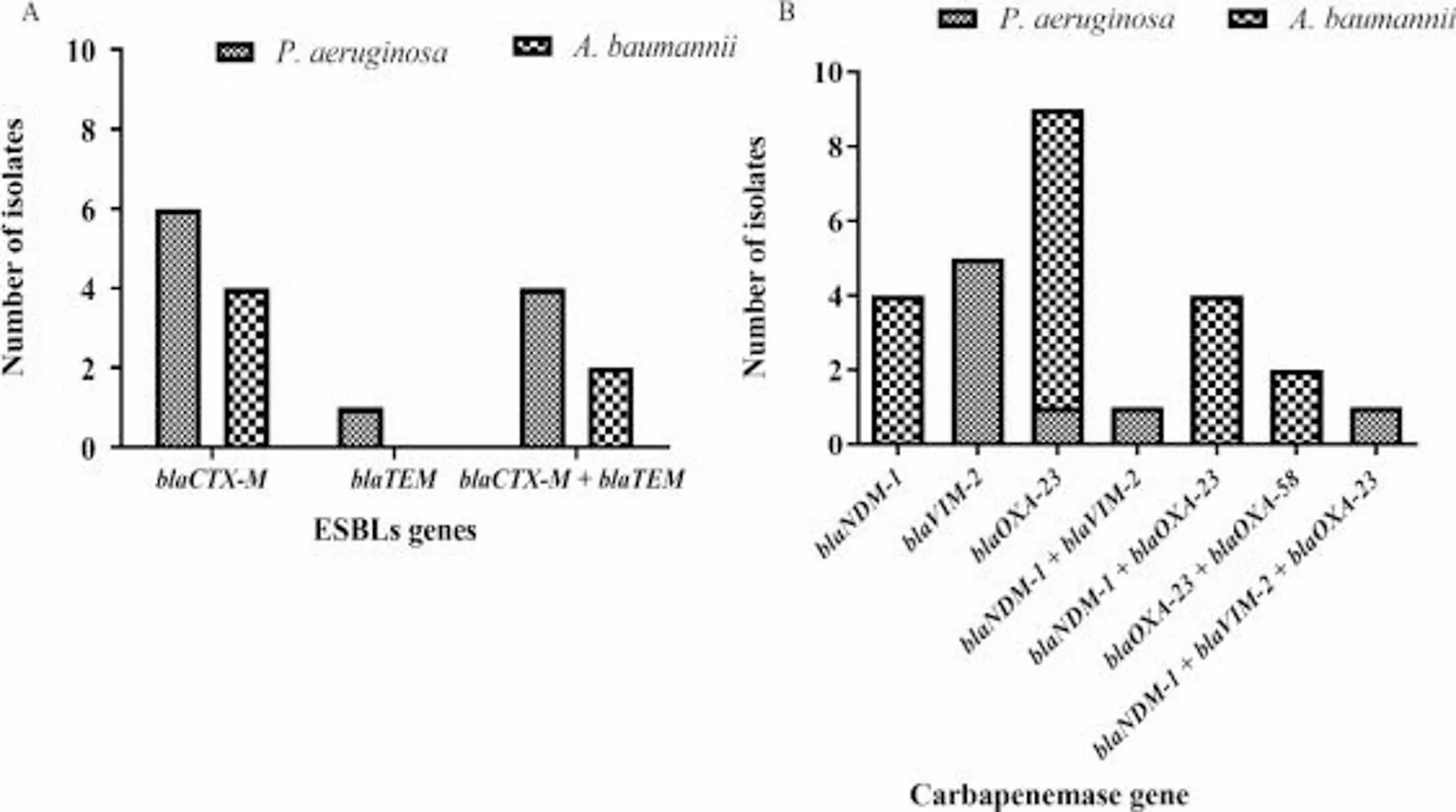
The presence of single genes or the occurrence of ESBL (**A**) and carbapenemase (**B**) genes in *P. aeruginosa* and *A. baumannii*

During the study period, 1,207 clinical specimens were collected and analyzed. Among the 1207 clinical specimens, 22.5% (95% CI: 20.2–24.8; *n* = 272/1207) showed bacterial growth. Out of the 272 total bacterial culture positives, 20.2% (95% CI: 15.4–25.0; *n* = 50/272) were gram-positive, and 79.8% (95% CI: 75.0-84.6; *n* = 217/272) were gram-negative (S-Fig. 1). Among the total gram-negative isolates, 14.7% (10.0-19.4; *n* = 32/217) were *A. baumannii*, and 12.5% (8.1–16.9; *n =* 27/217) were *P. aeruginosa*.

Distribution of NLF-GNB isolates, MDR, ESBL, carbapenem resistance, and MBL-producing isolates, with demographic data (age and sex) and hospital settings, is described in Table 1. Among the isolates, a higher prevalence of MDR, carbapenem resistance, ESBL, and MBL producers was detected in females than in males, but the difference was not statistically significant. In the age group category, a higher rate of MDR (37.8%; *n =* 17/45) was found in the 41–60-year age group, followed by the 21–40-year and older age groups (≥ 61 years). In contrast, carbapenem-resistant, ESBL-producing, and MBL-producing isolates were more prevalent in the younger age group (21–40 years), followed by the 41–60 years and ≥ 61 years age groups. The predominant MDR and carbapenem-resistant isolates were found in inpatient populations rather than in OPD patients. However, there was no statistically significant difference between these two groups of the population (Table 1).

**Table 1:** Distributions of NLF-GNB, MDR, ESBL, Carbapenem-resistant, and MBL among demographic groups

Variables	Total isolate (*n* = 59)		MDR (*n* = 45/59)		ESBL(*n* = 24/59)		Carbapenem Resistant (*n* = 37/59)		MBL (*n* = 28/59)	
Demographics	*P. aeruginosa* (*n* = 27)	*A. baumannii* (*n* = 32)	*P. aeruginosa* (*n* = 19)	*A. baumannii* (*n* = 26)	*P. aeruginosa* (*n* = 17)	*A. baumannii* (*n* = 7)	*P. aeruginosa* (*n* = 12)	*A. baumannii* (*n* = 25)	*P. aeruginosa* (*n* = 10)	*A. baumannii* (*n* = 18)
Gender (*n*/% at 95% CI)*										
Male	13/48.1 (95% CI: 29.2–67.0)	14/43.7 (95% CI: 26.5–60.9)	9/47.4 (95% CI: 22.4–70.4)	11/42.3 (95% CI: 23.3–61.3)	9/52.9 (95% CI: 28.9–76.9)	2/28.6 (95% CI: -4.4-61.6)	5/41.7 (95% CI: 13.7–69.7)	9/36.0 (95% CI: 17.0–55.0)	5/50.0 (95% CI: 19.0–81.0)	7/38.9 (95% CI: 15.9–61.9)
Female	14/51.9 (95% CI: 33.0-70.8)	18/56.3 (95% CI: 39.1–73.5)	10/52.6 (95% CI: 29.6–75.6)	15/57.7 (95% CI: 38.7–76.7)	8/47.1 (95% CI: 23.1–71.1)	5/71.4 (95% CI: 38.4-104.4)	7/58.3 (95% CI: 30.3–86.3)	16/64.0 (95% CI: 45.0–83.0)	5/50.0 (95% CI: 19.0–81.0)	11/61.1 (95% CI: 38.1–84.1)
Age Group (Years) (*n*/% at 95% CI)*										
≤ 20	2/7.4 (95% CI: -2.4-17.2)	5/15.6 (95% CI: 3.0-28.2)	1/5.3 (95% CI: -4.8-15.4)	5/19.2 (95% CI: 4.1–34.3)	1/5.9 (95% CI: -5.3-17.1)	1/14.3 (95% CI: -11.6-40.2)	-	4/16.0 (95% CI: 1.6–30.4)	-	4/22.2 (95% CI: 3.0-41.4)
21–40	6/22.2 (95% CI: 6.5–37.9)	13/40.7 (95% CI: 23.7–55.7)	5/26.3 (95% CI: 6.5–46.1)	10/38.5 (95% CI: 19.8–57.2)	5/29.4 (95% CI: 7.8–51.0)	4/57.1 (95% CI: 20.5–93.7)	3/25.0 (95% CI: 0.5–49.5)	10/40.0 (95% CI: 20.8–59.2)	3/30.0 (95% CI: 1.6–58.4)	8/44.4 (95% CI: 21.4–67.4)
41–60	9/33.3 (95% CI: 15.5–55.1)	9/28.1 (95% CI: 12.8–43.4)	7/36.8 (95% CI: 15.1–58.5)	8/30.8 (95% CI: 13.1–48.5)	6/35.3 (95% CI: 12.6–58.0)	2/28.6 (95% CI: -4.9-62.1)	4/33.3 (95% CI: 6.7–59.9)	8/32.0 (95% CI: 13.7–50.3)	4/40.0 (95% CI: 9.6–70.4)	3/16.7 (95% CI: -0.5-33.9)
≥ 61	10/37.1 (95% CI: 18.9–55.3)	5/15.6 (95% CI: 3.0-28.2)	6/31.6 (95% CI: 10.7–52.5)	3/11.5 (95% CI: 0.8–23.8)	5/29.4 (95% CI: 7.8–51.0)	-	5/41.7 (95% CI: 13.8–69.6)	3/12.0 (95% CI: 0.7–24.7)	3/30.0 (95% CI: 1.6–58.4)	3/16.7 (95% CI: -0.5-33.9)
Hospital Setting (*n*/%at 95% CI)*										
Inpatients	15/55.6 (95% CI: 36.9–74.3)	21/65.6 (95% CI: 49.1–82.1)	12/63.2 (95% CI: 41.5–85.5)	16/61.5 (95% CI: 42.8–80.2)	8/47.1 (95% CI: 23.4–70.8)	5/71.4 (95% CI: 37.9-104.9)	8/66.7 (95% CI: 40.2–93.2)	19/76.0 (95% CI: 59.3–92.7)	6/60.0 (95% CI: 30.0–90.0)	13/72.2 (95% CI: 55.7–88.7)
Outpatients	12/44.4 (95% CI: 25.7–63.1)	11/34.4 (95% CI: 17.9–50.9)	7/36.8 (95% CI: 15.1–58.5)	10/38.5 (95% CI: 17.1–54.5)	9/52.9 (95% CI: 29.2–76.6)	2/28.6 (95% CI: -4.9-62.1)	4/33.3 (95% CI: 6.8–59.8)	6/24.0 (95% CI: 7.3–40.7)	4/40.0 (95% CI: 10.0–70.0)	5/27.8 (95% CI: 11.3–44.3)

*MDR* Multidrug resistant, *ESBL *Extended-spectrum β-Lactamase, *MBL* Metallo-β-Lactamase

*A simple proportion-based prevalence CI at 95% level of significance calculation for *n* ≤ 50

In total, most of the isolates were obtained from sputum samples (40.7%, *n* = 24), followed by pus/wound swabs (20.3%, *n* = 12) (Table 2). We found a significant difference in the distribution of NLF-GNB isolates among different types of samples (*p* = 0.0253).

**Table 2:** Prevalence of *P. aeruginosa* and *A. baumannii* in different samples collected from inpatients and outpatients

Specimen	Inpatients (*n* = 36)		Outpatients (*n* = 23)		Total (*n* = 59) (*n*/% at 95% CI)*
	*P*. aeruginosa *(n* = 18*)* (*n*/%)	A. baumannii *(n* = 18*)* (*n*/%)	*P*. aeruginosa *(n* = 9*)* (*n*/%)	A. baumannii *(n* = 14*)* (*n*/%)	
Sputum	5 (27.8)	9 (50.0)	3 (33.3)	7 (50.0)	24 (40.7; 95% CI:28.1–52.5)
Pus/Wound swabs	6 (33.4)	2 (11.1)	3 (33.3)	1 (7.1)	12 (20.3; 95% CI:10.2–30.5)
Urine	5 (27.8)	-	2 (22.2)	2 (14.3)	9 (15.3; 95% CI: 6.8–22.0)
Blood	1 (5.5)	3 (16.7)	-	2 (14.3)	6 (10.2; 95% CI: 1.7–18.6)
Tips (ET, Catheter & Foleys)	1 (5.5)	4 (22.2)	1 (11.1)	2 (14.3)	8 (13.5;95% CI: 4.8–22.2)

*A simple proportion-based prevalence CI at the 95% level was calculated for the total isolated and not applicable for the subgroup data due to the small frequency

### Antimicrobial susceptibility test of the isolates

The majority of *P. aeruginosa* (*n =* 27*)* isolates were resistant to ceftazidime (63%), followed by gentamicin (59.3%) (Table S3). In the case of *A. baumannii* (*n* = 32), the highest level of resistance was observed against ceftriaxone (96.1%), followed by ceftazidime (93.7%), cefotaxime (87.5%), piperacillin (87.5%), ciprofloxacin (87.5%), piperacillin-tazobactam and cefepime (84.4%), gentamicin (81.3%), and carbapenem (78.1%).

### Carbapenem-resistant (CR) isolates

Among the 59 isolates, 62.7% (*n* = 37/59) were resistant to both meropenem and imipenem (carbapenem) (Table S3). The highest percentage of CR isolates was found in sputum samples (37.8%, *n* = 14/37), followed by pus/wound swabs (21.6%, *n* = 8/37) and tips, including ETs, catheters, and Foleys (6.2%, *n* = 6/37). The frequency of CR isolates significantly differed according to the specimen type (*p* = 0.0253) (Table S2).

Of the 37 CR isolates, the MIC values of meropenem for *P. aeruginosa* (*n* = 12) and *A. baumanni (n =* 25) ranged from 0.5 µg/mL to 512 µg/mL and from 0.5 µg/mL to 256 µg/mL, respectively. The MIC50 is higher in *A. baumanni* (64 µg/mL), and the MIC90 is higher in *P. aeruginosa* (256 µg/mL) (Table 3).

**Table 3:** Minimum inhibitory concentrations (MIC), MIC Range, MIC50, and MIC90 against meropenem (carbapenem) among the isolates

MIC value of Carbapenem (Meropenem) (µg/mL)	Organism	
	*P. aeruginosa* (*n* = 27)	*A. baumannii* (*n* = 32)
0.5	3	1
1	4	1
2	5	2
4	1	2
8	2	1
16	0	0
32	1	1
64	1	15
128	5	7
256	4	2
512	1	0
MIC Range	0.5–512	0.5–512
MIC_50_	8	64
MIC_90_	256	128

*MIC* Minimum Inhibitory Concentration, *µg/mL* Concentration of antibiotic in microgram per milliliter, *MIC50* MIC value at which growth was inhibited in 50% of isolates, *MIC90* MIC value at which growth was inhibited in 90% of isolates

### Prevalence of multidrug-resistant, ESBL-producing, and carbapenemase-producing NLF-GNB strains

Among the 59 isolates, 45 (76.3%) were confirmed as MDR isolates, among which 57.8% (*n* = 26/45) were *A. baumannii* and 42.2% (*n* = 19/45) were *P. aeruginosa*. Furthermore, of the 59 NLF-GNB isolates, 24 were confirmed to be ESBL positive, and *P. aeruginosa* (*n* = 17) was more common than *A. baumannii* (*n* = 7). Additionally, 62.7% (*n* = 37/59) were carbapenem-resistant, and a higher contribution was *A. baumannii* (*n =* 25/37). Whereas 75.7% (*n* = 28/37) carbapenem-resistant isolates were metallo-β-lactamase (MBL)-positive, comprising 64.3% (*n* = 18/28) were *A. baumannii* and 35.7% (*n* = 10/18) were *P. aeruginosa*. The frequencies of MDR, ESBL, and carbapenemase-producing NLF-GNB (MBL) strains are summarized in Table 4.

**Table 4:** Distributions of MDR, CR, ESBL, and MBL among NLF-GNB isolates

Features	Bacterial isolates (*n*/% at 95% CI)		Total (*n*/% at 95% CI)	*P*-value
	*P. aeruginosa* (*n =* 27)	A. *baumannii* (*n* = 32)	(*n* = 59)	
MDR	19 (70.4; 95% CI: 53.6–87.0)	26 (81.3; 95% CI: 68.4–93.2)	45 (76.3;95% CI:62.0-86.2)	0.328
ESBL	17 (62.9; 95% CI: 44.3–84.1)	7 (21.9; 95% CI:8.8–38.5)	24 (40.7; 95% CI:29.0-53.3)	*0.001
CR	12 (44.4; 95% CI: 25.0-68.3)	25 (78.1;95% CI:66.7–93.6)	37 (62.7; 95% CI:50.1–57.3)	*0.008
MBL	10 (37.0; 95% CI:18.2–56.4)	18 (56.3; 95% CI:41.3–71.2)	28 (47.5; 95% CI:33.2–59.3)	0.141

A simple proportion-based prevalence CI at 95% level of significance calculation for *n* ≤ 50

*MDR* Multidrug resistant, *ESBL *Extended-spectrum β-Lactamase, *CR* Carbapenem resistant, *MBL* Metallo-β-Lactamase

*Statistically significant (*P* < 0.05)

### Distribution of ESBL genes

A total of 24 isolates of the 59 were phenotypically ESBL-positive, where 66.7% (*n* = 16/24) *P. aeruginosa* and 29.2% (*n* = 7/24) *A. baumannii* were examined for the presence of ESBL-encoding genes via PCR. Among them, 25% (*n* = 6/24) of the isolates harbored both the *bla*_CTX−M_ and *bla*_TEM_ genes (four of the *P. aeruginosa isolates* and two of the *A. baumannii* isolates). A higher frequency of *bla*_CTX−M_ was detected in ESBL-positive *A. baumannii* (*n* = 5/7; 71.4%), whereas *bla*_TEM_ was more frequent in *P. aeruginosa* (25.0%; *n* = 4/16) (Table S4 and Fig. 1A).

### Distribution and co-occurrence of carbapenemase genes

All the isolates (*n* = 59) were examined for the presence of four carbapenemase genes, MBLs (*bla*_NDM−1_ and *bla*_VIM−2_), and OXA (*bla*_OXA−23_ and *bla*_OXA−58_), which are carbapenem resistance genes, via specific target primers described previously (Table S1).

Of the total CR isolates, 70.2% (*n* = 26/37) harbored at least one carbapenemase-encoding gene. Among these, 45.9% (*n* = 17/37) of the isolates contained metallo-β-lactamase (MBL) genes (Ambler class B), including *bla*_NDM−1_ in 10 isolates and *bla*_VIM−2_ in 7 isolates. Additionally, 48.6% (*n* = 18/37) of the isolates harbored OXA genes (Ambler class D), with *bla*_OXA−23_ present in 16 isolates and *bla*_OXA−58_ in 2 isolates. In the CRPA, *bla*_VIM−2_ was dominant (58.3%, *n* = 7/12), followed by *bla*_NDM−1_ and *bla*_OXA−23_ (16.7%, *n* = 2/12). Similarly, *bla*_OXA−23_ was highest (56.0%, *n* = 14/25) in CR-AB, followed by *bla*_NDM−1_ (32.0%, *n* = 8/25) and *bla*_OXA−58_ (8.0%, *n* = 2/25) (Table S4).

In total, 21.6% (*n* = 8/37) of the CR isolates coharbored more than one carbapenemase-encoding gene, whereas 47.4% (*n* = 18/37) of the isolates harbored a single gene. Specifically, four CR-AB isolates carried *bla*_NDM−1_ + *bla*_OXA−23_, two CR-AB isolates carried *bla*_OXA−23_ + *bla*_OXA−58_, and one CR-PA isolate tested positive for *bla*_NDM−1_ + *bla*_VIM−2_. Additionally, one CR-PA isolate was found to carry all three different carbapenemase-encoding genes (*bla*_NDM−1_ + *bla*_VIM−2_ + *bla*_OXA−23_) (Fig. 1B).

Although 11 isolates (four *P. aeruginosa* and seven *A. baumannii*) were phenotypically carbapenem-resistant, the associated genes were not detected. Interestingly, one of the carbapenem-susceptible isolates carried the *bla*_NDM−1_ and *bla*_OXA−23_ genes (Table S4).

## Discussions

In this study, 22.5% (*n* = 272/1207) of the total clinical specimens were bacterial culture positive; among them, 21.69% (*n* = 59/272) were *A. baumannii* and *P. aeruginosa*. The species identification of *P. aeruginosa* and *A. baumannii* was based on conventional phenotypic methods, with *bla*_*OXA−51*_ detection additionally used to support the identification of *A. baumannii*. Although these approaches are routinely employed in clinical microbiology laboratories, multiple identification criteria were applied to enhance diagnostic accuracy. However, the absence of comprehensive molecular confirmation may have resulted in occasional species misclassification, particularly among closely related non-fermenting Gram-negative bacteria. Consequently, the reported resistance phenotypes and genotypes should be interpreted cautiously. Future studies employing molecular identification methods, including sequencing-based approaches, are warranted to improve species-level accuracy and resistance surveillance.

CR-AB and CR-PA are the most common pathogens associated with nosocomial infections in Nepal [10]. *A. baumannii* and *P. aeruginosa* are frequently isolated from sputum and wound/pus samples. Carbapenem-resistant isolates were also more prevalent in those two samples, which may be due to their greater representation or could be linked to their ability to adapt to and resist specific environments where they persist. These results suggest that the dominance of antibiotic-resistant bacteria may differ in terms of survival mechanisms at different anatomical sites [27–30].

This investigation showed a high level of antibiotic resistance profile in clinical isolates to commonly used antibiotics. Approximately three-fourths of the *A. baumannii* isolates and half of the *P. aeruginosa* isolates were resistant to third-generation cephalosporins and carbapenem antibiotics. These comparative differences in resistance rates among the pathogens could be attributed to individual intrinsic resistance mechanisms [31]. Interestingly, our antimicrobial pattern was similar to several earlier observations of antibiotic resistance in Nepal [14, 32, 33].

Among the *A. baumannii* isolates, approximately 80% were carbapenem-resistant, which is a relatively lower prevalence than that reported in the research conducted by Joshi et al. (97.7%) in Kathmandu [14]. In contrast, another study conducted in tertiary care hospitals in Nepal reported a lower rate of CR-AB (51%) [34] than our study. Similarly, our neighboring countries, India (14.3%) [35] and Pakistan (22.0%) [36], also reported a low prevalence of CR-AB in clinical samples. In our findings, approximately 45% of *P. aeruginosa* isolates were carbapenem-resistant, which is similar to the results of a study conducted by Olalekan et al. in Nigeria [37]. However, in South Asian countries, Bangladesh reported approximately 40% carbapenem resistance, whereas Pakistan reported 65% carbapenem resistance in *P. aeruginosa* isolates [38]. These differences in outcomes could be the distributions of pathogens being influenced by microbial ecology, the hospital environment, infection control practices, patient care practices, coinfections, antibiotic use, study populations, geographic areas, and sample processes [11].

Moreover, we found that the range of MICs of meropenem was different for both *P. aeruginosa* (0.5–512 µg/mL) and *A. baumannii* (0.5–256 µg/mL), which could be related to the uncontrolled consumption of carbapenem antibiotics, constant exposure to antibiotics, and the accumulation of multiple resistant mechanisms in the clinical isolates [8].

The inpatient department showed an ideal environment for MDR and carbapenem-resistant organisms. These might be due to the vulnerable immunocompromised hosts, the use of invasive medical devices, and frequent antimicrobial therapy [39]. We found that 81.3% of *A. baumannii* strains were MDR, which was higher than a previous study conducted in Nepal. Studies conducted in various regions of Nepal reported 30.7%-75.9% rates of MDR *A. baumannii* [15, 34, 40]. In addition, 70.4% of the *P. aeruginosa* isolates were MDR in our study, which is higher than the findings of other studies in Nepal, and other studies reported the percentage ranged from 14.3% to 60.1% [15, 34, 40]. These results indicate that a higher prevalence of MDR isolates in our clinical setting, which creates limited treatment options for empirical treatment and higher mortality and morbidity rates, with respiratory tract infection, surgical site infection, and increased risk of spread in hospital environments.

This study also reported 40.7% prevalence of ESBL production among *A. baumannii* and *P. aeruginosa* isolates (Table 4). Notably, this study showed similarities with ESBL-producing *P. aeruginosa* from Kathmandu [34]. This study detected *bla*_TEM_ and *bla*_CTX−M_ genes among a subset of *A. baumannii* and *P. aeruginosa* isolates. The occurrence of *bla*_TEM_ and *bla*_CTX−M_ genes in *A. baumannii* was relatively lower than that in *P. aeruginosa*. This study revealed that the *bla*_CTX−M_ gene (66.7%) was higher than the *bla*_TEM_ gene (29.2%), and 25% of both genes coexisted. The previous studies have reported their presence in non-lactose fermenting Gram-negative bacteria, including *A. baumannii* and *P. aeruginosa* in India and Gaza [35, 41]. Although ESBL genes are predominantly associated with *Enterobacterales*, given that these ESBL variants are relatively uncommon in non-lactose fermenting bacteria and sequencing-based confirmation was limited, these findings are interpreted conservatively. Consequently, the precise sequence identity, genetic context, and functional expression of these amplicons remain unverified. Their detection in non-fermenting Gram-negative bacilli in this study might be due to horizontal gene transfer of ESBL-associated resistance genes in the hospital. However, horizontal acquisition of ESBL-associated genes represents a plausible explanation; this mechanism was not investigated in the present study.

We found that 75.7% (*n* = 28/37) of our carbapenem-resistant isolates produced MBL, among them two-thirds were *A. baumannii* (*n* = 18/28), and one-third were *P. aeruginosa* (*n* = 10/28). These results are well supported by a study conducted in Iran by Namaei et al. 2021 [42]. The previous studies have reported a lower prevalence of MBL-producing isolates in eastern Nepal [33, 43] and Kathmandu [34] than that observed in the present study. However, other studies reported that MBL production in *A. baumannii* ranges from 54 to 66.7% [33, 44], whereas in *P. aeruginosa* ranges from 8 to 45% in Nepal [32–34].

Among the CR isolates, 70.2% (*n* = 26/37) harbored at least one carbapenemase-encoding gene. Although 11 isolates (four *P. aeruginosa* and seven *A. baumannii*) were phenotypically carbapenem-resistant, the associated tested genes were not detected. These results might indicate that a significant amount of resistance is driven by structural changes (e.g., porin loss or altered efflux pumps) rather than the presence of specific resistance genes. PCR targets specific enzymes based on the primer design and will miss these structural changes, resulting in a higher phenotypic resistance rate than genotypic rate [45]. Moreover, some carbapenem-susceptible isolates identified via phenotypic detection may indeed be positive for carbapenemase genes by genotyping [46]. In the present study, we detected *bla*_ONDM−1_ and *bla*_OXA−23_ in one isolate and *bla*_CTX−M_ in two isolates of *P. aeruginosa* via PCR assays. Such genotype–phenotype discordance has been previously reported among carbapenemase-producing Gram-negative bacteria [21]. This discrepancy may be attributed to low-level or silent gene expression, absence of strong upstream promoter elements such as IS*Aba1*, gene mutations that impair enzyme function, or limitations in phenotypic susceptibility testing, lack of complementary resistance mechanisms (e.g., porin loss or efflux pump overexpression), or the presence of nonfunctional resistance genes [47]. However, these mechanisms were not investigated in the present study, and therefore their contribution to the observed discordance cannot be determined. Consequently, these interpretations are provisional and require validation in future studies incorporating gene expression analysis, sequencing-based characterization, and functional assays to elucidate the mechanisms underlying carbapenemase gene-positive phenotypically susceptible isolates.

Furthermore, *P. aeruginosa* and *A. baumannii* isolates harboring various carbapenemase resistance genes, including *bla*_NDM−1_, *bla*_VIM−2_, *bla*_OXA−23,_ and *bla*_OXA−58_, were identified in this study (Fig. 1B). The co-occurrence of *bla*_NDM−1_ and *bla*_VIM−2_ in *P. aeruginosa* and of *bla*_NDM−1_, *bla*_OXA−23,_ and *bla*_OXA−58_ in *A. baumannii* was reported. This finding agrees with various studies that have successfully reported the coexistence of antibiotic resistance genes among clinical isolates [48]. These multiple resistance genes found in the same isolates, as observed in our study, are indicative of the existence of multidrug-resistant pathogens and a variety of resistance mechanisms in clinical environments that could be responsible for the difficulty in treating and curing diseases.

The *bla*_VIM−2_ is the dominant gene in MBL-producing *P. aeruginosa* [49]. Similar results were found in our study; *bla*_VIM−2_ was also the predominant MBL gene among the CR-PAs co-occurring with *bla*_NDM−1_. These findings were also supported by previous studies conducted in Nepal [50, 51]. In contrast, a study from Nigeria revealed that the *bla*_NDM−1_ gene was more predominant than the *bla*_VIM−2_ gene among MBL-producing isolates [37].

More than half of the CR-AB isolates carried *bla*_OXA−23_, which was the predominant gene in our study. Other similar studies have also well documented *bla*_OXA−23_ as predominant among CR-ABs [36, 52]. A previous study in Nepal, CR-AB, has shown that the coexistence of *bla*_OXA−23_ with *bla*_NDM−1_ is significantly [14, 53]. This study also emphasized that *bla*_OXA−23_ and *bla*_ONDM−1_ could coexist in CR-AB more than in previous studies. Notably, 8% of the isolates had the *bla*_OXA−58_ gene, which actively coexists with *bla*_OXA−23_ and was reported in clinical CR-AB isolates in 2015 for the first time, in Nepal [53]. Moreover, very few CR-AB clinical isolates carry *bla*_OXA−58_ and are reported in the global context [53]. The co-occurrence of multiple resistance genes is a concern; it will increase the prevalence of carbapenem resistance in the future and limit therapeutic options for *A. baumannii* infections in a healthcare setting.

This study has some limitations. It was conducted at a single tertiary care hospital, which may limit the generalizability of the findings to other healthcare settings and geographic regions. The relatively small number of isolates reduced the statistical power for subgroup analyses and limited the robustness of comparisons between resistance gene groups. In addition, species identification of the isolates was based on phenotypic and biochemical characteristics, with *bla*_OXA−51_ PCR used to support *A. baumannii* identification. However, sequencing-based confirmation was not performed, and the possibility of species misidentification, particularly among closely related members of the *Acinetobacter calcoaceticus–baumannii* complex and other *Pseudomonas* strains, cannot be completely excluded. Moreover, the molecular analysis was limited to a selected panel of carbapenemase and β-lactamase genes and did not assess other potentially relevant resistance determinants. Similarly, mobile genetic elements and additional mechanisms contributing to antimicrobial resistance, including integrons, plasmids, transposons, efflux pumps, and porin alterations, were not investigated. As a result, the full genetic context, transmission dynamics, and molecular epidemiology of carbapenem resistance could not be comprehensively characterized. Future multicenter studies incorporating larger sample sizes, sequencing-based species confirmation, and comprehensive genomic analyses are warranted to provide a more complete understanding of antimicrobial resistance in these clinically important pathogens.

## Conclusion

Overall, this study revealed a high prevalence of MDR and carbapenem-resistant *P. aeruginosa* and *A. baumannii* isolates harboring multiple carbapenem resistance genes in the hospital setting of Nepal. This poses a significant challenge to the effective treatment and management of patients with currently available antibiotics in hospital settings, particularly in the developing countries of Nepal. Therefore, active surveillance and continuous monitoring of antibiotic resistance patterns in pathogens are essential to guide clinicians and healthcare providers in optimizing patient care and improving treatment outcomes.

## Supplementary Information

Supplementary Material 1. S1 Fig. 1: Frequencies of total bacterial species isolated from total samples. CONS: Coagulase-negative Staphylococcus spp. Table S1: PCR primers used for the detection of the ESBL and carbapenem-resistant genes in this study. Table S2: Distribution of carbapenem-susceptible and carbapenem-resistant isolates in various clinical specimens processed in this study. Table S3: Results of the antibiotic resistance profile by disk diffusion among P. aeruginosa and A. baumannii isolates. S-Table 4: Distribution of ESBL and Carbapenem-resistant genes in P. aeruginosa and A. baumannii isolates.

## Data Availability

All the information produced during this research work is presented within the manuscript and in the Supplementary file. Any queries regarding the research will be addressed by the corresponding author upon reasonable request.

## Abbreviations

AMR: Antimicrobial resistance
CLSI: Clinical Laboratory Standard Institute
CR: carbapenem resistance
CR-PA: carbapenem-resistant Pseudomonas aeruginosa
CR-AB: carbapenem-resistant Acinetobacter baumannii; CPase: carbapenemase
CONS: coagulase negative Staphylococcus
EDTA: ethylenediaminetetraacetic acid
ESBLs: extended-spectrum beta-lactamases
GNB: gram-negative bacilli
HAI: healthcare-associated infection
IRC: Institutional Review Committee
KMCTH: KIST Medical College and Teaching Hospital
KPC: Klebsiella pneumoniae carbapenemase
LMICs: low- and middle-income countries
MBLs: metallo-beta-lactamases
MDR: multidrug-resistant
MIC: minimum inhibitory concentration
NGS: next-generation sequencing
NLF: non-lactose fermenter
PCR: polymerase chain reaction
SPSS: Statistical Package for Social Sciences
WHO: World Health Organization
