# Supplementary material for: Phenotypic and molecular characterization of carbapenem resistance among clinical isolates of Pseudomonas aeruginosa and Acinetobacter baumannii
Source: BMC Microbiol. 2026 Jul 8;26:848. doi: 10.1186/s12866-026-05377-x (PMC13628859; doi:10.1186/s12866-026-05377-x)
Supplement: Supplementary file 1 [file 12866_2026_5377_MOESM1_ESM.docx]

**Carbapenem-resistant *Pseudomonas aeruginosa* and *Acinetobacter baumannii* clinical isolates from tertiary care in Nepal**

**Gopiram Syangtan^1^**^,2^, Laxmi Kant Khanal ^1, 3^, Srijana Bista ^2^, Arun Bahadur Chand ^4^, Prabin Dawadi ^2^, Binod Rayamajhee ^5^, Reshma Tuladhar^2^, Shiba Kumar Rai ^1,3,^ and Dev Raj Joshi ^2^

^1^Shi-Gan International College of Science and Technology (SICOST), Tribhuvan University, Kathmandu, Bagmati, Nepal.

^2^Central Department of Microbiology, Tribhuvan University, Kathmandu, Bagmati, Nepal

^3^Department of Microbiology, Nepal Medical College and Teaching Hospital, Kathmandu University, Kathmandu, Bagmati, Nepal

^4^Department of Microbiology, KIST Medical College and Teaching Hospital, Lalitpur, Bagmati, Nepal

^5^Department of Infection and Immunology, Kathmandu Research Institute for Biological Sciences (KRIBS), Lalitpur, Bagmati, Nepal

**Supplementary Information**

Figure: 01

Tables: 07

Pages: 06

**S-Figure 1:** Frequency of total bacterial species isolated from total specimens.

*CONS: Coagulase-negative *Staphylococcus* spp

**S-Table 1**: PCR primers used to detect ESBL and MBL genes in this study.

| **Genes** | **Primer Sequence** | **Amplicon size (bp)** | **Annealing Temperature** | **References** |
| --- | --- | --- | --- | --- |
| **ESBLs** | | | | |
| *bla*_CTX-M_ | F:5-GAGACAATAACCCTGGTAAAT-3 | 544 | 55 ˚C | (24) |
|  | R:5-AGAAGTAAGTTGGCAGCAGTG-3 |  |  |  |
| *bla*_TEM_ | F:5-TTTGCGATGTGCAGTACCAGTAA-3 | 459 | 56 ˚C | (25) |
|  | R:5- CGATATCGTTGGTGGTGCCATA-3 |  |  |  |
| **Carbapenemase** | | | | |
| *bla*_NDM-1_ | F:5-GGTTTGGCGATCTGGTTTTC-3 | 621 | 52 ˚C | (26) |
|  | R:5-CGAATGGCTCATCACGATC-3 |  |  |  |
| *bla*_VIM-2_ | F-5-ATGTTCAAACTTTTGAGTAAG-3 | 802 | 53 ˚C | (27) |
|  | R:5-CTACTCAACGACTGAGCG-3 |  |  |  |
| *bla*_OXA-23_ | F:5-GATCGGATTGGAGAACCAGA-3 | 501 | 52 ˚C | (28) |
|  | R:5-ATTCTGACCGCATTTCCAT-3 |  |  |  |
| *bla*_OXA-51_ | F:5-TAATGCTTTGATCGGCCTTG-3 | 353 | 57 ˚C | (28) |
|  | R:5-TGGATTGCACTTCATCTTGG-3 |  |  |  |
| *bla*_OXA-58_ | F:5-AAGTATTGGGGCTTGTGCTG-3 | 599 | 57 ˚C | (28) |
|  | R:5-CCCCTCTGCGCTCTACATAC-3 |  |  |  |

**S-Table 2:** Types of specimens processed in this study and number of GN-NLF-isolates and carbapenem-resistant.

| **Specimens** | **Total isolates (n= 59)** | | ***p*-value** | **Carbapenem-resistant isolates (n=37)** | | ***p*-value** |
| --- | --- | --- | --- | --- | --- | --- |
|  | *P. aeruginosa*  (n=27**/**%) | *A. baumannii*  (n=32**/**%) |  | *P. aeruginosa*  (n=12**/**%) | *A. baumannii*  *(*n=25**/**%) |  |
| Sputum | 8 (29.6) | 16 (50.0) | 0.006 | 2 (16.7) | 12 (48.0) | 0.004 |
| Urine | 7 (25.9) | 2 (6.3) |  | 3 (25.0) | 1 (4.0) |  |
| Blood | 1 (3.7) | 5 (15.6) |  | - | 5 (20) |  |
| Pus/wound swab | 9 (33.3) | 3 (9.4) |  | 5 (41.7) | 3 (12.0) |  |
| Tips (ET, Catheter and Foleys) | 2 (7.4) | 6 (18.7) |  | 2 (16.7) | 4 (16.0) |  |

**S-Table 3**: Results of antibiotics resistance profile by disk diffusion among non-lactose fermenter Grams negative isolates

| **Test Group** | **Antimicrobial agents** | **Bacterial isolates** | | | | | | ***P*-value** |
| --- | --- | --- | --- | --- | --- | --- | --- | --- |
|  |  | ***P. aeruginosa* (%)**  **(n=27)** | | | ***A. baumannii* (%)**  **(n=32)** | | |  |
|  |  | **S** | **I** | **R** | **S** | **I** | **R** |  |
| Penicillin | Piperacillin | 10 (37.0) | 3  (11.1) | 14 (51.9) | 3  (9.4) | 1  (3.1) | 28  (87.5) | 0.011 |
| β-lactam/ β-lactamase inhibitor combinations | Piperacillin-Tazobactam | 14 (51.9) | 6 (22.2) | 7 (25.9) | 4  (12.5) | 1  (3.1) | 27 (84.4) | 0.001 |
|  | Ceftazidime | 7  (25.9) | 3 (11.1) | 17 (63.0) | 2  (6.3) | - | 30 (93.7) | 0.001 |
| Cephalosporin | Cefepime | 12 (44.4) | - | 15 (55.6) | 3  (9.4) | 2  (6.3) | 27 (84.4) | 0.005 |
|  | Ceftriaxone | IR | | | 1  (3.1) | - | 31 (96.1) | NA |
|  | Cefotaxime | IR | | | 2  (6.3) | 2  (6.3) | 28 (87.5) | NA |
| Aminoglycosides | Gentamycin | 11 (40.7) | - | 16 (59.3) | 4  (12.5) | 2  (6.3) | 26 (81.3) | 0.026 |
|  | Amikacin | 15 (55.6) | 1  (3.7) | 11 (40.7) | 8  (25.0) | 1  (3.1) | 23 (71.9) | 0.05 |
| Fluoroquinolones | Ciprofloxacin | 11 (40.7) | 1  (3.7) | 15 (55.6) | 4  (12.5) | - | 28  (87.5) | 0.020 |
| Monobactam | Aztreonam | 11 (40.7) | 8 (29.6) | 8 (29.6) | IR | | | NA |
| Carbapenem | Meropenem | 15 (55.6) | - | 12 (44.4) | 6  (18.8) | 1  (3.1) | 25 (78.1) | 0.011 |
|  | Imipenem | 15 (55.6) | - | 12 (44.4) | 7 (21.9) | - | 25 (78.1) | 0.008 |
| *IR= Intrinsic Resistant and *NA= Not applicant | | | | | | | | |

**S-Table 4:** Distributions of MDR, CR, ESBL, and MBL among NLF-GNB isolates.

| **Features** | **Bacterial isolates** | | **Total (%)** | ***P*-value** |
| --- | --- | --- | --- | --- |
|  | *P. aeruginosa* (%) **(n=27)** | *A. baumannii* (%) **(n=32)** | (n=59) |  |
| **MDR** | 19 (70.4) | 26 (81.3) | 45 (76.3) | 0.328 |
| **ESBL** | 17 (63.0) | 7 (21.9) | 24 (40.7) | 0.001 |
| **Carbapenem Resistant** | 12 (44.4) | 25 (78.1) | 37 (62.7) | 0.008 |
| **MBL** | 10 (35.7) | 18 (64.3) | 28 (47.5) | 0.141 |

**S-Table 5:** Minimum Inhibitory Concentrations (MIC) of meropenem in CR isolates

| **Organisms** | **MIC value of Carbapenem (Meropenem) among Phenotypic CR**  **(****µg/mL)** | | | | | | ***P*-value** |
| --- | --- | --- | --- | --- | --- | --- | --- |
|  | **32** | **64** | **128** | **256** | **512** | **Total** |  |
| *P. aeruginosa* | 1 | 1 | 5 | 4 | 1 | 12 | 0.026 |
| *A. baumannii* | 1 | 15 | 7 | 2 | 0 | 25 |  |
| **Total (n)** | 2 | 16 | 12 | 6 | 1 | 37 |  |
| **MIC value of Carbapenem (Meropenem) among MBL-positive** | | | | | | | |
| *P. aeruginosa* | 0 | 1 | 4 | 4 | 1 | 10 | 0.073 |
| *A. baumannii* | 1 | 10 | 5 | 2 | 0 | 18 |  |
| **Total (n)** | 1 | 11 | 9 | 6 | 1 | 28 |  |

*MIC= Minimum Inhibitory Concentration * CR= Carbapenem Resistance

**S-Table 6:** Carbapenem resistant genes in NFL-GNB isolates from PCR assay.

| **Phenotypes and associated Genes** | **Carbapenem susceptible isolates**  **(n=22)** | | **Carbapenem resistant isolate**  **(n=37)** | | **Total (%)** |
| --- | --- | --- | --- | --- | --- |
|  | *P. aeruginosa*  (n=15) | *A. baumannii*  (n=7) | *P. aeruginosa*  (n=12) | *A. baumannii*  (n=25) |  |
| **MBL (Ambler Class B)** | | | | | |
| *bla*_NDM-1_ | 1 | **-** | 2 (16.7) | 8 (32.0) | 11 (18.6) |
| *bla*_VIM-2_ | **-** | **-** | 7 (58.3) | 0 | 7 (11.9) |
| **OXAs (Ambler Class D)** | | | | | |
| *bla*_OXA-23_ | 1 | **-** | 2 (16.7) | 14 (56.0) | 17 (28.8) |
| *bla*_OXA-58_ | **-** | **-** | 0 | 2 (8.0) | 2 (3.4) |

**S-Table 7.** Co-existence genes conferring resistance to β-lactams carbapenem among the carbapenem resistance isolates.

| Sample Code | MIC value | ESBLs | | | MBL | Carbapenem resistance genes | |
| --- | --- | --- | --- | --- | --- | --- | --- |
|  |  | Phenotype | Genes | | Phenotype | MBL-group | OXA-group |
| ***P. aeruginosa*** | | | | | | | |
| U-1026 | 256 | + | - | - | + | - | - |
| U-1216 | 64 | + | - | - | + | *bla*_VIM-2_ | - |
| 522 | 128 | - | - | - | + | - | - |
| 44 | 256 | + | *bla*_CTX-M_ | - | + | *bla*_NDM-1_ and *bla*_VIM-2_ | - |
| 625 | 256 | + | *bla*_CTX-M_ | - | + | *bla*_NDM-1_ and *bla*_VIM-2_ | *bla*_OXA-23_ |
| 814 | 128 | - | - | - | - | - | *bla*_OXA-23_ |
| 1435 | 128 | + | *bla*_CTX-M_ | - | + | *bla*_VIM-2_ |  |
| 1737 | 32 | + | *bla*_CTX-M_ | *bla*_TEM_ | - | - | - |
| C1 | 256 | - | - | - | + | *bla*_VIM-2_ | - |
| C2 | 128 | + | *bla*_CTX-M_ | - | + | *bla*_VIM-2_ | - |
| C11 | 128 | - | - | - | + | - | - |
| C12 | 512 | + | *bla*_CTX-M_ | *bla*_TEM_ | + | *bla*_VIM-2_ | - |
| ***A. baumannii*** | | | | | | | |
| 2226 | 64 | - |  | - | - | - | - |
| 1180 | 128 | - | - | - | + | - | *bla*_OXA-23_ |
| 1505 | 64 | - | - | - | - | - | - |
| 1274 | 128 | - | - | - | - | - | - |
| 1454 | 64 | - | - | - | - | - | - |
| 1347 | 64 | - | - | - | - | - | - |
| 1160 | 128 | + | *bla*_CTX-M_ | *bla*_TEM_ | + | *bla*_NDM-1_ | *bla*_OXA-23_ |
| 1503 | 64 | - | - | - | - | - | - |
| 1142 | 128 | - | - | - | + | *bla*_NDM-1_ | - |
| 1440 | 64 | - | - | - | + | - | *bla*_OXA-23_ |
| 592 | 64 | - | - | - | + | *bla*_NDM-1_ | - |
| 650 | 64 | + | *bla*_CTX-M_ | - | + | - | *bla*_OXA-23_ and *bla*_OXA-58_ |
| M694 | 64 | - | - | - | + | - | *bla*_OXA-23_ |
| A1 | 64 | - | - | - | + | *bla*_NDM-1_ | *bla*_OXA-23_ |
| A2 | 64 | - | - | - | + | - | *bla*_OXA-23_ |
| A4 | 128 | - | - | - | + | *bla*_NDM-1_ | - |
| P-510 | 256 | - | - | - | + | *bla*_NDM-1_ | *bla*_OXA-23_ |
| P-592 | 32 | - | - | - | + | - | *bla*_OXA-23_ |
| P-417 | 256 | - | - | - | + | *bla*_NDM-1_ | - |
| P-404 | 128 | + | *bla*_CTX-M_ | *bla*_TEM_ | - | - | *bla*_OXA-23_ |
| P-451 | 64 | + | *bla*_CTX-M_ | - | - | - | *bla*_OXA-23_ |
| U-832 | 64 | - | - | - | + | *bla*_NDM-1_ | *bla*_OXA-23_ |
| B-550 | 64 | + | *bla*_CTX-M_ | - | + | - | - |
| B-557 | 128 | - | - | - | + | - | *bla*_OXA-23_ and *bla*_OXA-58_ |
| 1524 | 64 | - | - | - | + | - | *bla*_OXA-23_ |
